# P-2075. Bridging the Gap: Early Intervention Services to Promote Retention in Care and Viral Suppression

**DOI:** 10.1093/ofid/ofaf695.2239

**Published:** 2026-01-11

**Authors:** Brianna Hohmann, Johnny Zakhour, Madison Patrus, Deena Gelfond, Amy Moon Kudlinski, Jamie G Joseph, Smitha Gudipati, Indira Brar

**Affiliations:** Henry Ford Health, Detroit, Michigan; Henry Ford Health, Detroit, Michigan; Henry Ford Health, Detroit, Michigan; Henry Ford Health, Detroit, Michigan; Henry Ford Health, Detroit, Michigan; Henry Ford Health/MSU, Royal Oak, Michigan; Henry Ford Health System, Detroit, MI; Henry Ford Hospital, Detroit, Michigan

## Abstract

**Background:**

Patients with HIV often face barriers to sustained care, especially due to social determinants of health (SDOH). Outreach and increased contact have been shown to improve follow-up. The Ryan White Early Intervention Services (EIS) program aims to support HIV care via referrals, education, and access. However, its impact on outcomes remains underreported. This study evaluated demographics of patients enrolled in EIS and outcomes in these patients.

**Table 1:**
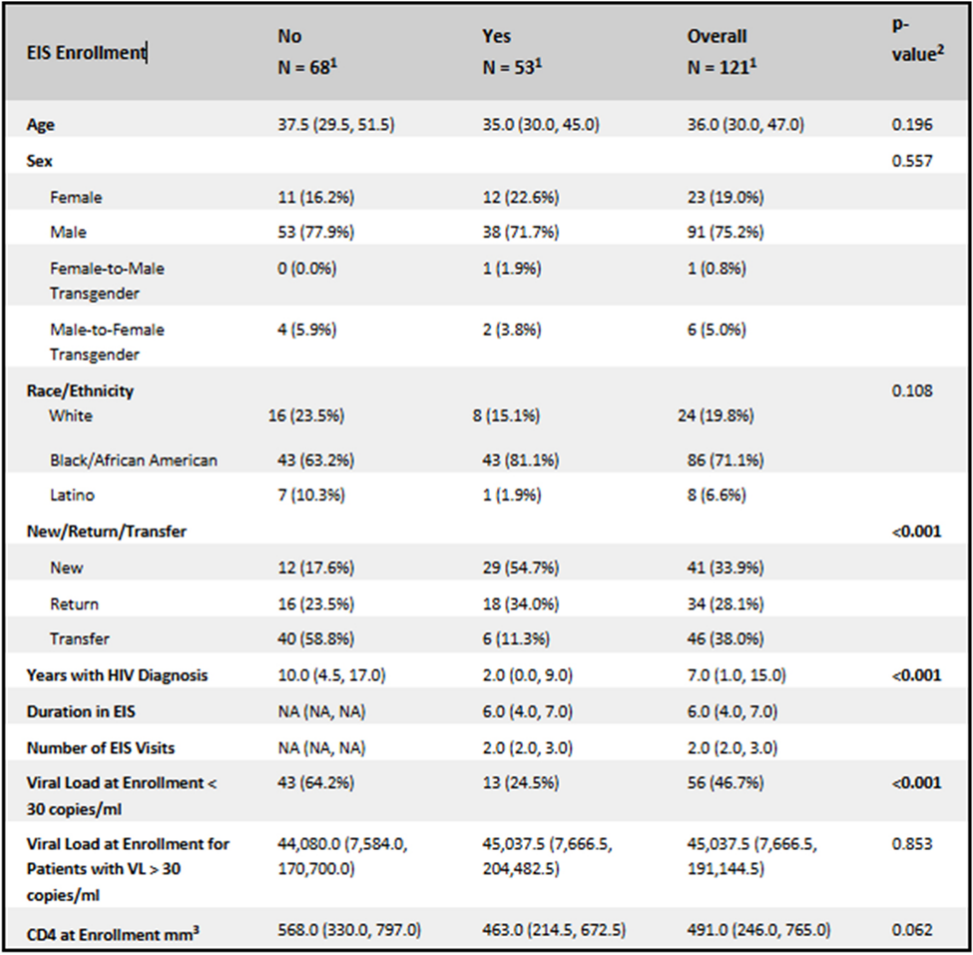
Baseline Demographics by EIS Enrollment 1Median (Q1, Q3); n (%) 2Wilcoxon rank sum test; Fisher's exact test; Pearson's Chi-squared test; Wilcoxon rank sum exact test

**Table 2:**
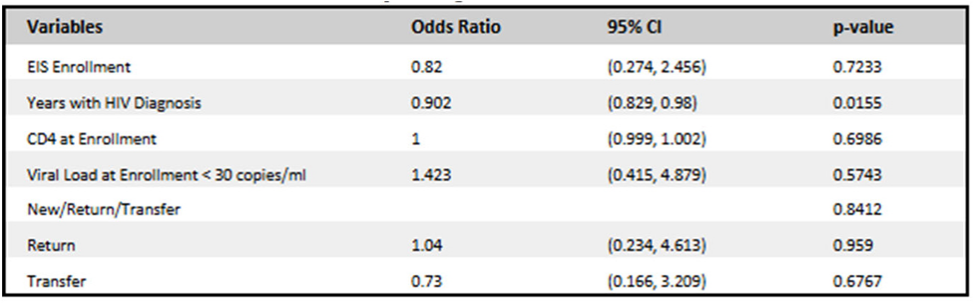
6-Month Follow-up Regression

**Methods:**

We conducted a retrospective chart review of adults newly enrolled in Ryan White services from January to December 2024. New diagnoses, return to care after more than 1 year, and transfers from another institution were reported. Patients enrolled in care coordination, lacking baseline data, or deceased were excluded. Descriptive statistics summarized demographics. Adjusted logistic regression assessed associations with follow-up and viral suppression.

**Table 3:**
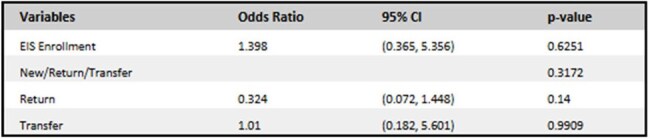
Viral Load Regression

**Results:**

Of 121 patients, 53 (43%) were in EIS. Most were male (71.7%) and Black (81.1%) (Table 1). New diagnosis was associated with EIS enrollment (p< 0.001). Suppressed viral load at baseline was more common in non-EIS patients (p< 0.001). The median duration in EIS was 6 months with median number of 2 visits (IQR 2.0–3.0). At 6 months, odds of follow-up were similar for EIS (57.3%) and non-EIS (54.9%) patients (p=0.723) (Table 2). Return patients had higher odds of following up at 6 months (aOR 1.04; 95% CI: 0.234-4.613). Viral suppression (VL < 30) was achieved in 60% of the 50 patients with follow-up data. Patients that enrolled in EIS experienced higher odds of achieving viral suppression (aOR1.398; 95% CI: 0.365-5.356). (Table 3) Return patients had lower odds of viral suppression (aOR 0.324; 95% CI: 0.072-1.448). Differences were not statistically significant.

**Conclusion:**

Our data suggests that EIS enrollment supports promoting viral suppression and retention in care. In addition to new patients, EIS efforts should focus on return patients and those without baseline viral suppression, particularly since these groups often have significant barriers to care. These findings highlight the need for proactive approaches to strengthen HIV care engagement, particularly for newly diagnosed or reengaging patients in a setting where SDOH play a crucial role in patient outcomes.

**Disclosures:**

Indira Brar, MD, Gilead: Advisor/Consultant|Gilead: Grant/Research Support|Gilead: Honoraria|ViiV Healthcare: Advisor/Consultant|ViiV Healthcare: Grant/Research Support|ViiV Healthcare: Honoraria

